# Measuring Socioeconomic Inequalities in Obesity among Korean Adults, 1998–2015

**DOI:** 10.3390/ijerph16091617

**Published:** 2019-05-08

**Authors:** Jongnam Hwang, Eun-Young Lee, Chung Gun Lee

**Affiliations:** 1 Division of Social Welfare & Health Administration, Wonkwang University, Iksan 54538, Korea; jonhwang416@gmail.com; 2 School of Kinesiology and Health Studies, Queen’s University, Kingston, ON K7L 3N6, Canada; eunyoung.lee@queensu.ca; 3 Institute of Sport Science, Department of Physical Education, Seoul National University, Seoul 08826, Korea

**Keywords:** obesity, socioeconomic inequality, concentration index, decomposition, Korea

## Abstract

Obesity is a prominent global public health challenge as its prevalence has grown. Even though the increase in prevalence of obesity in Korea has been relatively low, it is expected to continually increase in the next several years, leading to social and economic burdens. This study aimed to assess socioeconomic inequalities in obesity among Korean adults. Using nationally representative survey datasets, the concentration index (CI) and decomposition of the CI were used to capture and quantify obesity-related inequalities from 1998 to 2015. The results suggested that pro-poor inequalities in obesity existed in Korea, indicating that obesity was more concentrated among individuals with lower income. In a gender-stratified model, obesity was more concentrated among women with lower income and men with higher income, showing that the trend and magnitude of inequalities in obesity each vary by gender. The decomposition approach revealed that, over the past 17 years, the main contributors to the existing inequalities were higher education and higher income levels. These findings suggest that comprehensive and multifaceted interventions at the local and national levels should be considered to address the identified income- and education-related barriers with respect to obesity among Korean adults.

## 1. Introduction

Obesity is a growing public health challenge [1]. Over the past few decades, the prevalence of obesity has sharply increased in most countries [2]. A recent report from Organisation for Economic Co-operation and Development (OECD) highlights that the rapidly increasing overweight and obesity population among adults have become prevalent, with global estimated rates of 39% and 13%, respectively [3,4]. Korea has also been experiencing a similar trend in the prevalence of obesity among adults. While the rate of excess weight condition in Korea is one of the lowest among OECD countries, excess weight conditions have been steadily increasing in Korea over the past few years [5,6,7]. It has been estimated that over 30% of the adult population is overweight or obesity [8,9]. While this is still a relatively low prevalence, it is expected to continually increase, with indicators suggesting that the obesity rates will rise by a further 5% within in the next ten years [10].

The global increase of obesity poses considerable health-related problems. It has been well documented that obesity is a leading cause of noncommunicable diseases such as cardiovascular disease (CVD), hypertension, and some types of cancer [11,12]. Thus, diabetes and its related complications are also one of the most serious consequences of obesity in most industrialized countries [13]. In addition to its negative health outcomes, the obesity epidemic carries significant economic burdens. According to recent statistics, the economic impact of obesity amounts to $2 trillion annually, which is around 2.8% of the global Growth Domestic Product (GDP) [14]. The direct cost of obesity is responsible for approximately up to 7% of total healthcare costs in the U.S., and ranges from 1 to 5% in European countries [15]. In Korea it has been estimated that the annual costs attributable to obesity ranges from $170 million to $350 million, which is smaller than those of other developed countries, but still considerable [10]. Nonetheless, with growing rates of obesity in Korea, a constantly increasing obesity-related cost is anticipated over the next few decades [10].

A variety of factors can explain obesity. In general, obesity is closely linked to daily lifestyle, such as an increased intake of high-fat food and physical inactivity, as well as genetic predisposition [16]. A growing body of studies have highlighted that social and economic factors contribute to more to the current obesity epidemic than genetic predisposition based on existing evidence that genetic predisposition as a contributing factor is limited in explaining the rapid increase in most developed countries [1,17,18]. A major concern in these countries is that obesity might disproportionately affect socially and economically vulnerable groups, such as individuals with lower socioeconomic status (SES) and women in general [19,20]. Disproportionate obesity rates by SES could further worsen the socioeconomic gradients in health [21]. Previous studies in Canada, the U.S., and the UK support the occurrence of disproportionate rates of obesity by SES levels and gender [22,23,24]. For instance, an inverse relationship between SES and obesity is commonly observed, demonstrating that income and educational attainment are negatively associated with obesity [25,26]. In gender-specific studies of obesity, gender differences in obesity inequalities are considerable as women tend to be more sensitive to the inverse association between SES and obesity [27]. Some studies have suggested that the observed negative association between SES and excess weight condition can be attributed to unhealthy behaviors or lifestyle [20,28]. Several U.S. studies have found that people with higher educational attainment are less likely to have unhealthy lifestyles, and thus tend to be less obese than those with fewer years of schooling [29,30].

The existing studies in Korea have also demonstrated a pivotal role of socioeconomic factors in obesity [8,31,32]. While these studies have provided some evidence that socioeconomic differences in obesity are associated with other health behaviors (i.e., smoking food intake and physical activity), they failed to assess any trends or magnitude of obesity-related inequality. Furthermore, they did not decompose the extent of the observed inequalities in obesity so as to provide more information on the major contributors to the existing inequalities in obesity. Understanding the existence of socioeconomic inequalities in obesity and identifying the major contributing factors to these inequalities are crucial for developing effective and actionable policy suggestions to alleviate the existing inequalities across different socioeconomic groups over time.

The overall objective of this study was to provide a comprehensive picture of the socioeconomic inequalities in obesity over the past 17 years among Korea adults. The aim of this study was to assess the socioeconomic inequalities in obesity over the past 17 years and to identify the contributing factors to the observed inequalities and examine whether or not these contributing factors have changed. Given the importance of gender differences in obesity in the current literature, gender-specific analyses were provided.

## 2. Materials and Methods

### 2.1. Data Source

Data from the Korea National Health and Nutrition Examination Survey (KNHANES) from waves 1 (1998), 4 (2007–2009), and 6 (2013–2015) were analyzed. The KNHANES is a nationally representative survey for examining the health and nutritional statuses of Koreans and monitoring health-related risk factors as well as the prevalence of noncommunicable disease [33]. KNHANES is comprised of noninstitutionalized Koreans living in Korea who are sampled based on a multistage clustered probability design [33]. The KNHANES survey collects a wide range of information including sociodemographic status, health behaviors, quality of life, healthcare utilization, and health examination results [33]. In this study, we included respondents over the age of 18 who participated in KNHANES health examination (*n* = 8117 for wave 1, 16,536 for wave 4, and 12,917 for wave 6).

### 2.2. Variables

#### 2.2.1. Obesity

We used individuals’ Body Mass Index (BMI), derived from height and weight measurements collected during physical health examination (Weight (kg)/Height (m)^2^). The following cut off values were used to determine obesity; obesity (BMI ≥ 25), otherwise (BMI ≤ 24.9) [34,35,36]. This dichotomy approach for measuring the CI was used in previous studies [27,37].

#### 2.2.2. Socioeconomic Status

In order to measure the degree of inequality in obesity, equivalized income, as calculated based on self-reported annual household by the square root of the number of household members, was used. Income is one of the main SES indicators, and it has been commonly used for Concentration Index (CI) and decomposition analyses [38].

#### 2.2.3. Other Variables for Decomposition Analysis

For our decomposition models, other variables for obesity were selected based on determinants of health, in particular, excess weight conditions from previous studies. These variables included sociodemographic factors such as sex, age, marital status, educational level, employment condition, place of residence, and self-rated health reflecting an individual’s health.

### 2.3. Statistical Analyses

To measure the socioeconomic inequalities in overweight and obesity conditions for wave 1 (1998), wave 2 (2007–2009), and wave 3 (2013–2015), the CI was calculated for each wave of the KNHANES. The CI is a widely-used measurement for assessing health and health care inequality in the areas of health economics and policy research [39]. After obtaining the CIs, decomposition approaches were applied to quantify the sociodemographic factors contributing to observed inequalities and their changes in contributions to sociodemographic factors over the past years, with a focus on obesity in Korean adults over time.

#### 2.3.1. Concentration Index

The CI is defined as twice the area between the 45-degree line (also called the line of equality) and a concentration curve, where the individuals are placed by income levels, and the cumulative ranking of each individual is plotted against the cumulative share of health outcomes. The CI is typically bound between −1 to +1, where a positive (negative) value emerges when the outcome variable is concentrated among the relatively rich (poor). The CI was calculated using Equation (1):

(1) $$C\;=\;\frac{2*cov\left(y_{i}*r_{i}\right)}{\mu }$$

where *y* is the health variable, *r* is the fractional rank in the income distribution, and μ is the mean of the health variable. It has been discussed that applying the CI method for dichotomized outcome variables has limitations, as the CI is bound differently according to different mean values of the outcome variable. In order to rectify this issue, the C can be normalized by multiplying (1 − mean of the outcome variable), the result of which is referred to as normalized CI in this study, following prior studies [40]. Alternatively, the C needs to be modified by multiplying 4μ/(*b* − *a*), where *a* is a lower bound and *b* is an upper bound of the binary outcome because the CI needs to reveal the same magnitude of inequality when calculated on the basis of both health and ill health variables [41].

#### 2.3.2. Decomposition of the CI

The decomposition method was as described in previous studies [37,42,43]. The basic idea of decomposing the CI involves disaggregating the observed inequalities by calculating the inequality and elasticity of each factor included in the decomposition model. The decomposition analyses were calculated by Equation (2):

(2) $$C_{\mathrm{total}}\;=\;\sum_{k}\left(\frac{\beta_{k}{\bar{x}}_{k}}{\mu }\right)C_{k}+\frac{CG_{k}}{\mu }$$

where the index *K* refers to the regressor included in the underlying equation, *β_κ_* is the coefficient for each health determinant, ${\bar{x}}_{k}$ is the mean of each regressor, *C_κ_* is the CI for each individual regressor, and μ is the mean of the health variable under consideration. *CG_ε_* is the generalized C for the residual from the underlying regression. All analyses were conducted using STATA v. 15 (StataCorp, College Station, TX, USA).

## 3. Results

**Figure 1:**
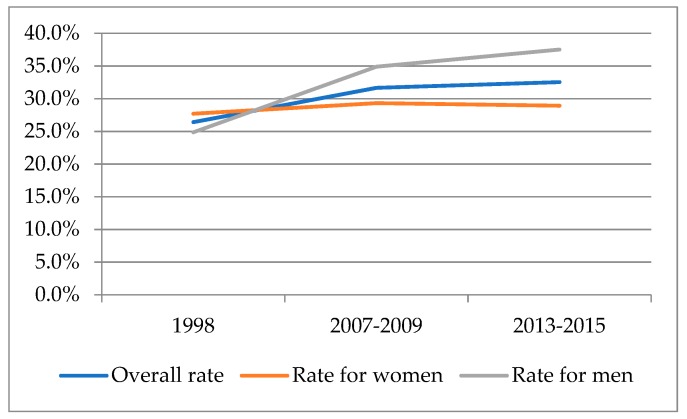
Rates of obesity among Korean adults from the Korea National Health and Nutrition Examination Survey (KNANES).

The descriptive statistics showed that the rate of obesity increased from 26.4% to 32.5% over the 17-year period considered. The data also showed gender variations. As illustrated in Figure 1, the rates of obesity increased for both Korean women and men. Meanwhile, obesity has been consistently more prevalent in Korean men than women over the same period.

Table 1 and Table 2 present income-related inequalities in obesity among Korean adults and by gender as measured by Concentration Indexes (CI). The results showed that obesity was more concentrated among the rich in 1998, but the direction of CI changed to negative values. This observed change in the CIs shows that obesity became more concentration among lower income groups in the 2000s. The results of gender-stratified analyses indicate that the CI has steadily shrunken in Korean men, approaching “0”, the line of equality. The values of the CIs were in the “pro-rich” direction over the past years, suggesting that the observed inequalities favor higher income groups. In women, the CIs for obesity consistently had negative values, indicating a higher concentration of obesity in the poor. Thus, the magnitude of the inequalities has increased from −0.070 (wave 1) to −0.186 (wave 6). Because the outcome variable is binary (i.e., obesity vs. non-obesity), the Erreygers correction of the CI was applied. The results showed the same direction of inequality, but the magnitude of the inequality slightly decreased as presented in both Table 1 and Table 2.

Table 3 demonstrates the contributions of each sociodemographic factor to the observed inequalities in obesity in Korean adults. A positive (negative) elasticity indicates that an increase in an explanatory variable increases (decreases) the probability of being obesity. The Concentration Index for each variable represents if the factor is more concentrates among rich or poor individuals. The positive (negative) CI means the factor is concentrated among high-income (low-income) individuals. The positive contribution of each variable to the observed inequality indicates that the income distribution of each factor and the association between each factor and obesity lead to increased probability of being obesity among the rich. In other words, a positive contribution from a certain factor implies that the observed inequality could be reduced by x% if the factor were distributed equally across different income groups or if the factor was not associated with obesity. In 1998, the largest contribution to the CI for obesity came from income, particularly from the higher income group. The age 36–50-year group was the second-largest contributor. In 2007–2009, higher educational attainment groups were the largest contributor to the observed pro-poor inequality in obesity, and they remained the largest contributor in 2013–2015, followed by higher income groups.

Table 4 and Table 5 report the results from decomposition analyses for Korean women and men, respectively. Higher educational attainment explains the largest fraction of the obesity inequalities for women in all waves. If there were no contribution from the completion of high school and college or more groups, the degree of obesity inequality would have been approximately 28% and 46% smaller (closer to zero) in the first wave. The education contribution springs from education being both unequally distributed and correlated with obesity. The contributions for the age variables were large in wave 1, while the total contribution diminished in wave 6. Regarding men, contributions from income reduced as the degree of inequality for obesity lessened over time. In the meantime, age became the largest contributor to the observed pro-rich inequality. The age 65 or more group was concentrated in the relatively poor in wave 6, and the obesity elasticity is negative.

## 4. Discussion

Using the CI and decomposition of the CI methods, this study aimed to assess socioeconomic inequalities in obesity, and quantified each contribution from sociodemographic factors to the observed inequalities over the past years. By employing the Concentration Index (CI) approach, this study reveals that inequalities in obesity were in the pro-rich direction in 1998 but changed to the pro-poor direction after 2007 to 2009. In general, obesity is more common among individuals of higher SES in low- and middle-income countries, whereas the reverse relation is seen in high-income countries [2,44]. Ample evidence suggests that socioeconomic inequalities in obesity are persistent in other developed countries, although the magnitude of inequalities differs [13,21,23,37]. As compared to other high-income countries, where obesity is commonly seen more often in the poor, it is notable that obesity was more common in high-income groups in late 1990s, but that the pattern has changed to in favor of the poor since the 2000s.

A plausible explanation for the changes in inequality patterns could be attributed to the changes towards to a more obesogenic environment that makes individuals with lower SES more vulnerable to obesity over a 17-year period [45]. Accompanied with economic growth and lifestyle changes, the lower SES group is more susceptible to unhealthy physical, economical, and social environments [28,46]. It has been reported that Korean society has been experiencing residential segregation and social stratification by socioeconomic status [47,48]. Therefore, individuals with lower income tend to live in impoverished neighborhoods with a lack of accessibility to healthier food, less walkable environments, and limited exercise facilities [49,50]. A recent study focusing on dwellers in Seoul, the capital city of the country, shows that the rate of obesity varies across the 25 districts of Seoul; specifically, obesity is less prevalent in affluent neighborhoods with better physical environments [51]. In addition, knowledge and social/cultural values could contribute to this observed change in inequalities in obesity. Higher SES groups, mostly higher income groups, have an educational advantage for understanding the health value of an appropriate weight, diet, and physical activities, and in the more effective application of knowledge about health to everyday life [52]. In fact, a higher concentration of obesity among higher income or education groups has been reported in the U.S. and Canada, and those with high SES are more attuned to maintain and support healthy lifestyle [27,37,53]. As the Korean society developed and progressed more toward other high-income countries, public perception toward obesity has similarly changed to that of other developed countries, representing that obesity may not be an acceptable and healthy condition with perspectives of health and beauty, mostly for the better-off [54]. This implies, along with our findings, that investment and resource allocation for lower SES groups as the main target group needs to be implemented as a higher concentration of obesity in the poor appears while the rate of obesity steadily increases over time in Korea [46,55].

This study further indicates that the trends of inequalities in obesity differ by gender, pointing to consistent pro-rich inequalities in men while pro-poor inequalities persist in women. This might be a result of cultural and social value on ideal body weight by gender. Excess weight traditionally symbolizes high social class, power, and physical prowess, while preference for thinness exist among Korean women immersed in the stigma and bias faced by obesity [56]. Korean women with higher SES may have more resources to stay thin and more access to obtaining better knowledge regarding healthy weight control behaviors as compared to their lesser SES peers [57]. Several studies have also highlighted similar patterns of gender-related inequalities in obesity in different countries [58]. Studies found that lower SES women, particularly those with lower income or education level, were more likely to be obesity in some European countries and other high-income countries, whereas there was a higher concentration of obesity among men with higher SES [2,27,37,59]. Accompanied by cultural values and social norms, different patterns of physical activity in disadvantaged SES groups could translate into different trends of inequalities by gender. For instance, men with lower SES are inclined to engage in unskilled jobs, which typically require more physically demanding work [59]. Nonbehavioral factors may be associated with gender-related inequalities in obesity. A plausible mechanism was suggested that lower SES women tend to be more vulnerable than their counterparts with the same SES to negative psychosocial and material exposures through their life course, and this leads possible influences on obesity over disturbances in physiological stress systems [60,61,62,63]. Considering the steadily increasing obesity rate, health policy should invest more on worse-off women and better-off men to tackle the obesity epidemic in Korea.

The results of our decomposition analysis assure that income and educational level are considerable factors to the observed inequalities over a 17-year period. The results also point to the urgency of understanding the mechanisms linking inequalities and obesity among Korean adults. A previous study suggested three possible pathways as to how SES and obesity can be associated with each other [55]: a structural pathway that emphasizes a causal relation of inequality on residential environment and poverty [55]. Individuals dwelling in impoverished neighborhoods face increased risks of obesity because of a limited supply of affordable healthy food, unfriendly walking environment, higher crime rates, and lack of an adequate facility for physical activity [64]. Lack of social capital and social cohesion is another casual pathway [65]. It is well-documented that the better-off are more inclined to invest and maintain social capital and social cohesion, which can in turn contribute to better health outcomes, including healthy weight control [65,66]. Based on this close link between social capital and health condition by income level, economically disadvantaged groups may have limited resources and support to invest and interact with their social network. Taken together, this generates more psychological stress and emotional instability, ultimately leading to less attentiveness to weight-related issues [55]. At the macro-level, an absence of social and health policy to eliminate health inequalities may accelerate the existing inequalities in obesity [67]. For instance, a study on an association between income inequality and obesity highlighted a possible relation between high rates of obesity and an absence of public health insurance in the U.S., which is the only country that does not provide universal health coverage for their citizens among the OECD countries [55]. Although all residents in Korea are eligible for National Health Insurance, there are still doubts as to whether any policy programs at the local and central government levels to tackles socioeconomic inequalities in health including obesity have been attempted.

In addition to SES, age is also a pivotal factor to understanding why socioeconomic inequalities in obesity exist in Korea. The results from the decomposition analyses suggest that contributions from older age groups to the observed inequalities became greater, implying that older adults can experience increasing burdens of economic deficiency and obesity-related health issues. Considering the rapid aging of the population and the disproportionately increasing rate of poverty among old adults in Korea [68], policy interventions need to be developed to facilitate weight control for older adults.

Taken together, our findings suggest that obesity-related policies focused on improving the circumstances of the most disadvantaged groups need to be considered. This is because those who are disadvantaged may not be effectively influenced by traditional public health interventions focused on individual level behavior change and which do not tackle its root causes [17]. Two policy approaches to reduce inequalities in obesity that warrant consideration are the following; (1) universal policies providing an additional support and investment on the worse off, targeting for men with higher SES and women with lower SES, respectively, and (2) proportionately universal policies that provide progressively greater benefit as one descends down the socioeconomic spectrum, which ultimately contribute to reducing overall prevalence of obesity in Korea [69].

While the calculation and decomposition of the CIs allow one to observe trends in obesity inequalities and factors contributing to the observed inequalities, some limitations have been identified. The CI is a descriptive approach, so it does not provide any supporting evidence that income or other socioeconomic factor is a determinant of obesity but suggest to what extent income and obesity are associated to one another by comparing with the poor and the rich. In addition, the decomposition approach does not provide any causal pathway between socioeconomic factors and obesity; however, it reveals extra factors that are simultaneously correlated with the existing relation between income and obesity. Furthermore, it is worthwhile to note that the decomposition we applied in this study only explains the degree of variation in health or health-related outcome as this approach is one-dimensional, focusing on health rather than the covariance between health and rank. Future study may need to consider the application of alternative decomposition methods for the inequality index that attempts to overcome the raised concerns in the literature [70].

## 5. Conclusions

Although the prevalence of obesity in Korea is relatively low as compared to that in other high-income countries, the findings of this study suggest that obesity-related inequalities exist among Korean adults. In particular, obesity become more concentrated in the worse off, and the results from gender-stratified analyses demonstrate that obesity is more commonly observed in poor women and rich men. Additionally, the decomposition analyses reveal higher income and educational level as major contributors to the observed inequalities that favor the better-off, in addition to age. To alleviate socioeconomic inequalities in obesity, comprehensive and multifaceted interventions need to be considered so as to tackle the observed inequalities, particularly the higher concentrations of obesity in certain socioeconomic groups in Korea.

## Figures and Tables

**Table 1:** The Concentration Index (CI) for obesity in Korean adults, 1998−2015.

KNHANES Wave	Wave 1 (1998)	Wave 4 (2007–2009)	Wave 6 (2013–2015)
Concentration Index	0.018	−0.041	−0.076
Erreygers correction	0.014	−0.035	−0.067
SE	0.011	0.007	0.007
95% CI	−0.010–0.047	−0.060–−0.022	−0.096–−0.056

**Table 2:** The Concentration Index (CI) for obesity in Korean women and men, 1998–2015.

	KNHANES Wave	Wave 1 (1998)	Wave 4 (2007–2009)	Wave 6 (2013–2015)
Women	Concentration Index	−0.070	−0.175	−0.186
	Erreygers correction	−0.056	−0.145	−0.152
	SE	0.014	0.009	0.010
	95% CI	−0.074–−0.023	−0.200–−0.150	−0.212–−0.159
Men	Concentration Index	0.137	0.126	0.048
	Erreygers correction	0.103	0.114	0.045
	SE	0.017	0.009	0.009
	95% CI	0.093–0.181	−0.017–0.030	0.019–0.077

**Table 3:** Results from decomposition analysis for Korean adults, KNHANES 1998–2015.

Variables	Wave 1 (1998)				Wave 4 (2007–2009)				Wave 6 (2013–2015)			
	Elasticity	CI	Contribution	%	Elasticity	CI	Contribution	%	Elasticity	CI	Contribution	%
Gender (ref. Male)												
Female	0.133	−0.005	−0.001	−3.85	−0.386	−0.007	−0.067	−6.65	−0.490	−0.007	0.003	−4.36
Age (ref. 19–34)												
35–49	0.108	0.140	0.015	82.53	0.077	0.243	0.019	−45.81	0.050	0.227	0.011	−14.92
50–64	0.090	−0.157	−0.014	−77.21	0.098	−0.030	−0.003	7.26	0.072	0.041	0.003	−3.85
65+	0.011	−0.401	−0.004	−23.90	0.019	−0.463	−0.009	21.65	0.030	−0.428	−0.013	17.09
Education level (Ref. Elementary)												
Middle school	0.005	−0.050	0.000	−1.36	−0.015	−0.095	0.001	−3.48	−0.018	−0.171	0.003	−3.98
High school	−0.078	0.105	−0.008	−44.44	−0.093	0.120	−0.011	27.38	−0.090	0.083	−0.008	9.89
College or more	−0.063	0.307	−0.019	−105.06	-0.087	0.347	−0.030	73.39	−0.102	0.302	−0.031	40.62
Income (Ref. Q1)												
Q2	0.019	−0.225	−0.004	−23.72	0.010	−0.200	−0.002	5.04	−0.005	−0.207	0.001	−1.37
Q3	0.015	0.183	0.003	14.97	−0.012	0.147	−0.002	4.34	−0.005	0.154	−0.001	0.99
Q4	0.032	0.660	0.021	116.36	−0.001	0.633	−0.001	1.67	−0.022	0.626	−0.014	18.51
Employment (ref. White collar)												
Blue collar	0.022	0.159	0.004	19.14	0.010	0.125	0.001	−3.01	0.005	0.088	0.000	−0.52
Nonworking	−0.038	−0.137	0.005	28.72	−0.041	−0.132	0.005	−13.23	−0.002	−0.110	0.000	−0.33
Unemployment	−0.032	−0.095	0.003	16.75	−0.025	−0.112	0.003	−6.82	−0.021	−0.150	0.003	−4.11
Marital status (ref. Married or partnered)												
Singled	−0.040	−0.108	0.004	23.56	0.016	0.069	0.001	−2.70	0.029	0.071	0.002	−2.72
Place of residence (ref. Metro-Seoul region)												
Non-metro Seoul region	0.000	0.118	0.000	0.03	0.014	0.090	0.001	−3.11	−0.002	0.066	0.000	0.21
Self-rated health												
Fair	−0.024	0.059	−0.001	−7.60	0.010	0.045	0.000	−1.15	0.041	0.009	0.000	−0.46
Bad	−0.019	−0.190	0.004	19.62	0.032	−0.225	−0.007	17.53	0.034	−0.220	−0.007	9.84
Sum			0.006				−0.099				−0.046	
Residual (Total C-Sum)			0.012				0.058				−0.030	
Total CI for obesity			0.018				−0.041				−0.0760	

**Table 4:** Results from decomposition analysis for Korean women, KNHANES 1998–2015.

Variables	Wave 1 (1998)				Wave 4 (2007–2009)				Wave 6 (2013–2015)			
	Elasticity	CI	Contribution	%	Elasticity	CI	Contribution	%	Elasticity	CI	Contribution	%
Age (ref. 19–34)												
35–49	0.070	0.146	0.010	−14.54	0.069	0.254	0.017	−9.97	0.054	0.240	0.013	−6.93
50–64	0.122	−0.200	−0.024	34.99	0.109	−0.068	−0.007	4.23	0.110	0.024	0.003	−1.41
65+	0.052	−0.373	−0.019	27.80	0.079	−0.466	−0.037	21.08	0.092	−0.452	−0.042	22.41
Education level (Ref. Elementary)												
Middle school	0.023	0.004	0.000	−0.14	−0.009	−0.016	0.000	−0.09	−0.014	−0.121	0.002	−0.93
High school	−0.121	0.166	−0.020	28.65	−0.126	0.179	−0.023	12.86	−0.118	0.138	−0.016	8.76
College or more	−0.097	0.334	−0.032	46.37	−0.134	0.370	−0.050	28.23	−0.168	0.316	−0.053	28.47
Income (Ref. Q1)												
Q2	0.038	−0.216	−0.008	11.62	−0.005	−0.214	0.001	−0.61	−0.022	−0.214	0.005	−2.51
Q3	0.022	0.162	0.004	−5.17	−0.041	0.147	−0.006	3.46	−0.021	0.152	−0.003	1.75
Q4	0.022	0.653	0.015	−20.96	−0.030	0.635	−0.019	10.70	−0.057	0.624	−0.035	19.00
Employment (ref. White collar)												
Blue collar	0.028	0.171	0.005	−6.89	0.020	0.093	0.002	−1.05	−0.001	0.076	0.000	0.03
Nonworking	−0.008	−0.228	0.002	−2.53	0.000	−0.222	0.000	−0.03	0.001	−0.174	0.000	0.12
Unemployment	0.020	−0.021	0.000	0.59	0.059	−0.038	−0.002	1.29	0.002	−0.090	0.000	0.09
Marital status (ref. Married or partnered)												
Singled	−0.067	−0.140	0.009	−13.44	0.110	0.099	0.011	−6.23	0.084	0.104	0.009	−4.70
Place of residence (ref. Metro-Seoul region)				23.56								
Non-metro Seoul region	0.015	0.144	0.002	-3.18	0.000	0.093	0.000	0.00	-0.006	0.070	0.000	0.21
Self-rated health												
Fair	−0.021	0.086	−0.002	2.64	0.005	0.065	0.000	−0.18	0.003	0.011	0.000	−0.02
Bad	−0.018	−0.178	0.003	−4.65	0.038	−0.222	−0.008	4.78	0.023	−0.199	−0.005	2.51
Sum			−0.057				−0.120				−0.124	
Residual (Total C-Sum)			−0.013				−0.055				−0.062	
Total CI for obesity			−0.070				−0.175				−0.186	

**Table 5:** Results from decomposition analysis for Korean men, KNHANES 1998–2015.

Variables	Wave 1 (1998)				Wave 4 (2007–2009)				Wave 6 (2013–2015)			
	Elasticity	CI	Contribution	%	Elasticity	CI	Contribution	%	Elasticity	CI	Contribution	%
Age (ref. 19–34)												
35–49	0.071	0.134	0.010	7.01	0.043	0.230	0.010	7.91	0.014	0.210	0.003	6.06
50–64	−0.001	−0.108	0.000	0.07	0.049	0.019	0.001	0.75	−0.003	0.066	0.000	−0.44
65+	−0.055	−0.448	0.024	17.87	−0.064	−0.463	0.030	23.47	−0.053	−0.400	0.021	43.89
Education level (Ref. Elementary)												
Middle school	0.016	−0.112	−0.002	−1.34	0.012	−0.192	−0.002	−1.87	0.001	−0.238	0.000	−0.66
High school	0.063	0.040	0.003	1.85	0.036	0.044	0.002	1.27	0.025	0.014	0.000	0.72
College or more	0.054	0.286	0.015	11.24	0.040	0.321	0.013	10.20	0.050	0.285	0.014	29.31
Income (Ref. Q1)												
Q2	−0.012	−0.233	0.003	2.03	0.016	−0.185	−0.003	−2.42	0.002	−0.199	0.000	−0.83
Q3	−0.010	0.205	−0.002	−1.53	0.008	0.151	0.001	0.94	−0.002	0.159	0.000	−0.74
Q4	0.044	0.670	0.030	21.57	0.018	0.632	0.011	8.82	−0.002	0.631	−0.001	−2.74
Employment (ref. White collar)												
Blue collar	0.037	0.151	0.006	4.08	0.011	0.179	0.002	1.63	0.009	0.117	0.001	2.24
Nonworking	−0.006	−0.094	0.001	0.44	−0.060	−0.084	0.005	4.04	0.016	−0.085	−0.001	−2.74
Unemployment	0.001	−0.273	0.000	−0.26	−0.015	−0.294	0.004	3.52	−0.011	−0.280	0.003	6.35
Marital status (ref. Married or partnered)												
Singled	−0.093	−0.046	0.004	3.12	0.097	0.028	0.003	2.20	0.116	0.027	0.003	6.38
Place of residence (ref. Metro-Seoul region)												
Non-metro Seoul region	−0.023	0.107	−0.002	−1.77	0.031	0.092	0.003	2.24	−0.003	0.062	0.000	−0.37
Self-rated health												
Fair	−0.019	0.029	−0.001	−0.40	0.012	0.023	0.000	0.22	0.067	0.006	0.000	0.80
Bad	−0.029	−0.208	0.006	4.47	0.014	−0.222	−0.003	−2.44	0.034	−0.252	−0.009	−17.78
Sum			0.094				0.076				0.034	
Residual (Total C-Sum)			0.043				0.050				0.015	
Total CI for obesity			0.137				0.126				0.048	
